# GSK3β and aging liver

**DOI:** 10.18632/aging.100060

**Published:** 2009-06-22

**Authors:** Jingling Jin, Guo-Li Wang, Lubov Timchenko, and Nikolai A Timchenko

**Affiliations:** ^1^Huffington Center on Aging and Department of Pathology, Baylor College of Medicine, Houston, TX 77030, USA; ^2^Department of Molecular Physiology and Biophysics, Baylor College of Medicine, Houston, TX 77030, USA

**Keywords:** aging, cyclin D3, C/EBP, GSK3, liver, proliferation

## Abstract

The loss of regenerative capacity of tissues is one of the major characteristics of aging. Liver represents a powerful system for investigations of mechanisms by which aging reduces regenerative capacity of tissues. The studies within last five years revealed critical role of epigenetic silencing in the inhibition of liver proliferation in old mice. These studies have shown that a number of cell cycle proteins are silenced in livers of old mice by C/EBPα-HDAC1-Brm complex and that old liver fails to reduce the complex and activate these genes in response to proliferative stimulus such as partial hepatectomy. The complex modifies histone H3 on the promoters of c-myc and FoxM1B in the manner which prevents expression of these genes. Despite this progress, little is known about mechanisms by which aging causes this epigenetic silencing. We have recently discovered signal transduction pathways which operate upstream of the C/EBPα-HDAC1-Brm complex.  These pathways involve communications of growth hormone, GSK3β and cyclin D3. In addition to the liver, GH-GSK3β-cyclin D3 pathway is also changed with age in lung, brain and adipose tissues. We suggest that other age-associated alterations in these tissues might be mediated by the reduced levels of GSK3β and by elevation of cyclin D3.  In this review, we summarize these new data and discuss the role of such alterations in the development of aging phenotype in the liver and in other tissues.

## Complexity of the mechanisms which reduce regenerative capacity of the liver

Age-associate inhibition of liver proliferation has been described over 50 years ago [2] and has been the subject of intensive investigations especially during last 6 years.  The initial studies have been focused on the investigations of the role of individual genes in the inhibition of liver proliferation [3,4,5].   However, several recent papers have found that the inhibition of liver proliferation in old mice is associated with formation of multi-protein C/EBPα-Brm complexes in nucleus [6,7] and multi-protein complexes of RNA binding protein CUGBP1 with translation initiation factor eIF2 in cytoplasm [8,9,10]. Following studies showed that these complexes alter transcription and translation in livers of old mice [10-13].   It has been later shown that the activation of CUGBP1 in livers of old mice leads to the translational elevation of a chromatin remodeling protein histone deacetylase 1, HDAC1, which joins the C/EBPα-Brm complex and silences promoters of the cell cycle genes [10].  In addition to the intracellular alterations, Rando's group has found that systemic environment of young animals reduces C/EBPα-Brm complex and corrects liver proliferation [7].  We have recently found that glycogen synthase 3β, GSK3β, is a key enzyme which regulates these pathways in the liver and that the decline of GSK3β with age causes inhibition of liver proliferation via stabilization of cyclin D3 and following changes in transcription and translation [1].  This review discusses age-associated mechanisms of inhibition of liver proliferation in the light of this recent finding.

## GSK3β regulates transcription and translation in the liver via control of cyclin D3

**Figure 1.**
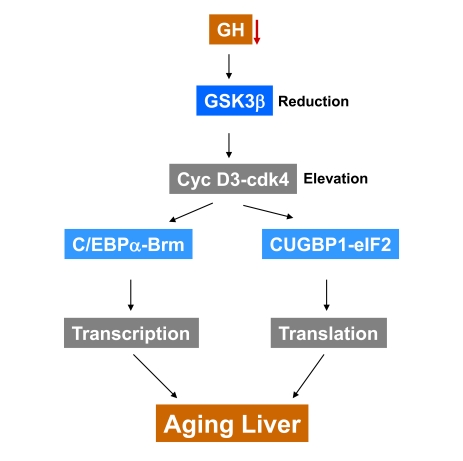
A hypothesis for the role of reduction of GSK3β in development of aging phenotype in the liver. GSK3β triggers degradation of cyclin D3 in livers of young mice.  The age-associated decline of growth hormone and GSK3β leads to the stabilization of cyclin D3 and to formation of transcriptional repressor C/EBPα-Brm and translational activator CUGBP1-eIF2 complexes.  We suggest that the appearance of these two comp-lexes in the liver might change global transcription and translation leading to the development of aging phenotype in the liver.

GSK3β is a ubiquitously expressed multifunctional serine/threonine protein kinase originally identified as a key regulator of insulin-dependent glycogen synthesis [14,15].  GSK3β phosphorylates a number of substrates which are involved in embryonic development, protein synthesis, mitosis, and survival [16-19])**.  **In addition to these activities, GSK3β has been shown to support cell proliferation and liver regeneration [20,21]. Little is known about the mechanisms by which GSK3β regulates cell proliferation. It has been shown that GSK3β inhibits Wnt signaling through stabilization of β-catenin and that this pathway is involved in development of cancer [22,23]. The essential role of active GSK3β in cell survival has been shown in the studies of GSK3β-null mice which die during embryogenesis due to liver degeneration caused by widespread hepatocyte apoptosis [24].  Several papers showed that inappropriate modulation of GSK3β activity plays critical role in the age-related pathologies such as Alzheimer's disease, noninsulin-dependent diabetes mellitus, inflammation, and cancer [21,25,26,27,28]**.  **We have recently identified mechanisms by which GSK3β regulates biological functions of the liver and mechanisms by which aging reduces GSK3β in the liver and alters two levels of regulation of gene expression: transcription and translation through the reduction of GSK3β [1].  In livers of young mice, GSK3β phosphorylates cyclin D3 and controls cyclin D3-cdk4 on relatively low levels. Our data show that GSK3β is reduced with age and that the age-associated decline of GSK3β leads to stabilization of cyclin D3 and following accumulation of transcriptional C/EBPα-Brm and translational CUGBP1-eIF2 complexes (Figure 1). We suggest that the alterations in epigenetic repression of genes and alterations in translation of certain proteins result in development of aging phenotype in the liver.  What target genes might be affected by these two multi-protein complexes?  The C/EBPα-Brm complex binds to and represses the promoters of S-phase specific genes [29].  We have shown that the CUGBP1-eIF2 complex increases translation of two proteins, C/EBPβ and HDAC1, in livers of old mice. The biological consequences of the elevation of C/EBPβ and HDAC1 are discussed in our recent review [30].  In summary, our findings placed GSK3β in the network which regulates transcription and translation in the liver and emphasized the role of decline of GSK3β in development of aging phenotype in the liver.  In agreement with our findings, Seo et al have recently found that the inactivation of GSK3β by specific inhibitors, by dominant negative mutant GSK3β-K85A or by siRNA effectively induces senescence phenotype in human liver-derived Chang cells [31]. Taken together our results and these data, we suggest that the decline or inactivation of GSK3β play a critical role in the development of senescence phenotype in the liver.

## GSK3β-cyclin D3 pathway is altered in brain, lung and adipose tissues of old mice

Systemic environment of young mice corrects proliferation of the liver and regeneration of skeletal muscle in old mice [7]. Because growth hormone (GH) regulates cyclin D3 in the liver through GSK3β and because it is one of the components of the systemic environment which is reduced with age, we suggested that GH might also regulate GSK3β-cyclin D3 pathway in other tissues. Given the fact that the target of cyclin D3/cdk4, C/EBPα, is expressed at high levels in brain, lung and adipose tissue, we have examined the GSK3β-cyclin D3 pathway in these additional tissues. Similar to alterations in the liver, we found the age-associated reduction of GSK3β and elevation of cyclin D3 in all tested tissues.  It is interesting that the administration of GH restores GSK3β-cyclin D3 pathway in these tissues [1]. Although our studies were focused on the liver and on two known targets of cyclin D3, C/EBPα and CUGBP1, the age-associated alterations of GSK3β and cyclin D3-cdk4 presumably affect several other targets in different tissues. The future studies are required for understanding of all biological consequences of alterations in GSK3β-cyclin D3 pathway. It would be interesting to examine additional tissue-specific targets of both cyclin D3/cdk4 and GSK3β in tissues of old mice. In skeletal muscle, cyclin D3-cdk4 interacts with MyoD [32] and potentially the age-associated elevation of cyclin D3-cdk4 might re-program expression of genes in skeletal muscle through MyoD.  It is also interesting to determine if the reduction of GSK3β in tissues of old mice affects pathways which are dependent on GSK3β and independent on cyclin D3/cdk4.  Since the cytoplasmic target of cyclin D3-cdk4, CUGBP1, is expressed in all tissues, it would be important to examine the age-associated alterations in the translational targets of the CUGBP1-eIF2 complex. The significance of this pathway is discussed in our recent review [30].   In summary, our new data suggest that the age-associated alteration of the GSK3β-cyclin D3 pathway is one of the critical events in the development of aging phenotype in the liver and perhaps in other tissues.
